# Influence of Light of Different Spectral Compositions on the Growth, Photosynthesis, and Expression of Light-Dependent Genes of Scots Pine Seedlings

**DOI:** 10.3390/cells10123284

**Published:** 2021-11-24

**Authors:** Pavel Pashkovskiy, Vladimir D. Kreslavski, Yury Ivanov, Alexandra Ivanova, Alexander Kartashov, Alexander Shmarev, Valeriya Strokina, Vladimir V. Kuznetsov, Suleyman I. Allakhverdiev

**Affiliations:** 1 K.A. Timiryazev Institute of Plant Physiology, Russian Academy of Sciences, Botanicheskaya Street 35, 127276 Moscow, Russia; ivanovinfo@mail.ru (Y.I.); aicheremisina@mail.ru (A.I.); botanius@yandex.ru (A.K.); vlkuzn@mail.ru (V.V.K.); 2 Institute of Basic Biological Problems, Russian Academy of Sciences, Institutskaya Street 2, 142290 Pushchino, Russia; vkreslav@rambler.ru (V.D.K.); shurik_bx_04@mail.ru (A.S.); strokina.93@mail.ru (V.S.)

**Keywords:** photomorphogenesis, *Pinus sylvestris*, light of various spectral composition, photosynthesis, chlorophyll fluorescence, gene expression, pigment content

## Abstract

Varying the spectral composition of light is one of the ways to accelerate the growth of conifers under artificial conditions for the development of technologies and to obtain sustainable seedlings required to preserve the existing areas of forests. We studied the influence of light of different quality on the growth, gas exchange, fluorescence indices of Chl *a*, and expression of key light-dependent genes of *Pinus sylvestris* L. seedlings. It was shown that in plants growing under red light (RL), the biomass of needles and root system increased by more than two and three times, respectively, compared with those of the white fluorescent light (WFL) control. At the same time, the rates of photosynthesis and respiration in RL and blue light (BL) plants were lower than those of blue red light (BRL) plants, and the difference between the rates of photosynthesis and respiration, which characterizes the carbon balance, was maximum under RL. RL influenced the number of xylem cells, activated the expression of genes involved in the transduction of cytokinin (Histidine-containing phosphotransfer 1, *HPT1*, Type-A Response Regulators, *RR-A*) and auxin (Auxin-induced protein 1, *Aux/IAA*) signals, and reduced the expression of the gene encoding the transcription factor phytochrome-interacting factor 3 *(PIF3*). It was suggested that RL-induced activation of key genes of cytokinin and auxin signaling might indicate a phytochrome-dependent change in cytokinins and auxins activity.

## 1. Introduction

The quality of light is an important factor in regulating plant growth and development during ontogenesis, including germination, photomorphogenesis, flowering induction, etc. At the beginning of ontogenesis, most plants are forced to vegetate under shading conditions while growing underneath taller plants, which also leads to a decrease in the quality of light. Green and far-red light (FRL) predominate under the forest canopy because light in the red and blue ranges of the spectrum is effectively absorbed by the chlorophyll of taller plants. This forces the seedlings of most woody plants to adapt to indifferent light qualities [1]. For example, plants growing under a forest canopy acclimatize to a low red:far red (R:FR) ratio, which causes shoot elongation [1]. It was previously shown that blue light (BL), on the contrary, inhibits shoot growth [2,3]. This leads to plants with a high BL level and a high R:FR ratio growing low but with an increased leaf surface, which in turn affects the intensity of photosynthesis [4].

The observed changes in the global climate under all forecast scenarios suggest a decline in coniferous species in the temperate zone of Europe [5]. In this regard, the development of technologies for obtaining sustainable seedlings is required to preserve the existing areas of coniferous forests. Varying the spectral composition of light can be one of the simplest ways to accelerate the growth of seedlings under artificial conditions [6]. However, to select the optimal spectral composition of light under artificial light conditions, it is necessary to know how the light of different spectral ranges affects the growth and photosynthetic parameters of seedlings of coniferous plants.

Plants have several types of photoreceptors that respond to changes in environmental light conditions. Under natural light, plants are simultaneously exposed to light of different wavelengths, resulting in cross-signaling of light between multiple photoreceptors. Phytochromes are among the most characterized photoreceptors. They are RL and FRL sensors and they regulate many developmental processes, including seed germination and hypocotyl growth [1]. Other well-known receptors, the cryptochromes, perceive light in the blue and UV ranges of the spectrum. They are involved in the growth processes and de-etiolation of seedlings and are also involved in circadian rhythms [7]. Photoreceptors have been studied in detail in *Arabidopsis thaliana*. They include five phytochromes (PHYA to PHYE) and two major cryptochromes (CRY1 and CRY2). In gymnosperms, PHYN is orthologous to PHYA of angiosperms, while PHYO is orthologous to PHYC [8]. In addition, gymnosperms have PHYP, which genetically occupies an intermediate position between the PHYB of *Oryza sativa* and the PHYE of *Arabidopsis thaliana* [9]. From the experiments carried out by Clapham et al., 2002, it was found that pine and spruce react to the RL/FRL ratio differently than angiosperms, which confirms the uniqueness of the phytochrome system of gymnosperms [10].

In addition to the quality of light, plant growth is also affected by hormonal balance [11]. The lighting conditions cause the level of hormones in plants to change, which leads to a change in photosensitivity. For example, exogenous hormones can stimulate plant growth by acting as mediators in the processes of light signal transduction [12]. In turn, light by photoreceptors regulates the metabolism of various hormonal signals. Thus, PHYA affects the metabolic pathways of gibberellins and indoleacetic acid [11], as well as key components of light signaling, such as the transcriptional factors (TFs): PIF3, PIF4, and HY5 [13]. The main phytohormones are associated with light-mediated growth regulation [14,15,16], while the stimulation of cell growth involves auxins and cytokinins [17,18,19]. The light of different spectral compositions affects the activity of endogenous hormones through the regulation of secondary metabolism; for example, blue light promotes the accumulation of flavonoids, which in turn affects the polar transport of auxin [20].

The aim of this work was to understand how the conditions of light of different spectral composition affect the growth, morphometric, and photosynthetic characteristics of *Pinus sylvestris* seedlings. At the same time, special attention was given to the possible relationships of the processes of growth, photosynthesis, and respiration with the intensity of expression of the main genes encoding proteins of photosystems, and the genes involved in light and hormonal signalling of *Pinus sylvestris* plants under light of various spectral quality. The obtained results can be used to create artificial lighting systems in forest nurseries to accelerate the cultivation of planting material, and they also have applications in biotechnology.

## 2. Materials and Methods

### 2.1. Plant Materials and Experimental Design

**Figure 1:**
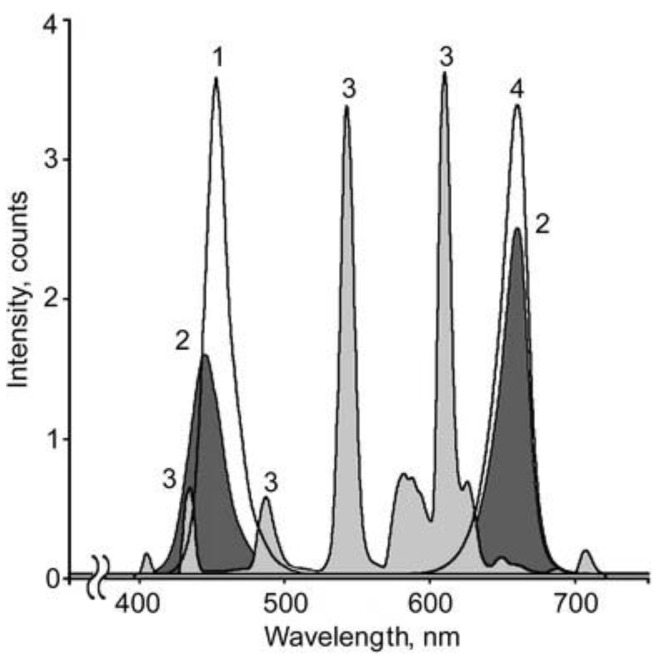
Emission spectra of the light sources used in the experiments. Spectra of blue light (BL) with a peak at 450 nm (1), blue + red (BRL) with two maxima at 445 nm and 660 nm (2), white fluorescent lamps (WFL) with a set of peaks in the visible spectral region (3), and red light (RL) with a peak at 660 nm (4).

Seeds of Scots pine (*P. sylvestris* L.) were collected in the Bryansk region (Bryansk, Russia) from high-productivity pine stands in complex forest types. Seeds were germinated and grown in hydroculture on individual substrates in polypropylene cartridges filled with 1% agar bungs in individual boxes of the climatic chamber under red (maxima of 660 nm), blue (maxima of 450 nm), red + blue (maxima of 660 and 450 nm) LEDs, as well as under fluorescent lamps (58 W/33–640, white fluorescent lamps (Philips, Pila, Poland), 130 ± 10 μmol (photons) m^−2^ s^−1^) for 6 weeks. (Figure 1).

After seed coat rupture and cotyledon expansion, the seedlings were transferred to a nutrient solution [21]. The seedlings were cultivated in 6 L plastic trays (171 seed beds per tray) in a growth chamber that provided a constant air temperature of 24 ± 2 °C and a 16 h photoperiod. The nutrient solutions were constantly aerated and renewed once a week. During the week, a constant volume of the nutrient solution was maintained by adding distilled water.

The fresh biomass of the roots and needles was determined using an analytical balance (Scout Pro SPU123, Ohaus Corporation, Parsippany, NJ, USA) with an accuracy of 1 mg, after which the samples were fixed in liquid nitrogen and stored at −70 °C until biochemical analyses. The fixation was carried out under the conditions of the light in which the plants grew without exposure to another light.

### 2.2. Pigment Contents

The contents of chlorophyll *a* (Chl *a*) and *b* (Chl *b*) and total carotenoids (Car) in pigment extracts of all studied needles were determined spectrophotometrically in 80% acetone [22].

### 2.3. Measurements of CO_2_ Gas Exchange

The photosynthetic rate (P_n_) was determined in a closed system under light conditions using an LCPro + portable infrared gas analyser from ADC BioScientific Ltd. (United Kingdom) connected to a leaf chamber. The CO_2_ uptake per leaf area (μmol m^−2^s^−1^) was determined. The rate of photosynthesis of the leaves in the second layer from the top was determined at a saturating light intensity of 1000 μmol (photons) m^−2^ s^−1^. Previously, we recorded the light curves in the interval of intensities from 0 to 1200 μmol (photons) m^−2^ s^−1^. The light intensities in the region from 600 to 1200 μmol (photons) m^−2^ s^−1^ were saturated. After measuring the rate of photosynthesis, the light was turned off, and the rate of dark respiration was measured.

### 2.4. Determination of Photochemical Activity

Fluorescence parameters characterizing the state of the photosynthetic apparatus were calculated on the basis of induction fluorescence curves obtained using data from the JIP test, which is usually used to evaluate the state of PSII. Chlorophyll (Chl) fluorescence induction curves (OJIP curves) were recorded with the setup Plant Efficiency Analyser (Handy-PEA, Hansatech Instruments Ltd., London, UK). For the JIP test, OJIP curves were measured under illumination with blue light at an intensity of 3500 μmol (photons) m^−2^ s^−1^ for 1 s.

On the basis of induction fluorescence curves (OJIP curves), the following parameters, which characterize the PSII photochemical activity, were calculated: F_v_/F_m_, the PSII maximum quantum photochemical yield, and PI_ABS_, the PSII performance index [23,24]. Here, F_v_ is the value of variable fluorescence, equal to the difference between F_m_ and F_0_; F_0_ is the minimum amplitude of fluorescence (F), and F_m_ is the maximum amplitude of fluorescence. For calculation of the PI_ABS_, the following formula was used:

PI_ABS_ = (F_v_/F_m_)/(M_0_/V_j_) × (F_v_/F_0_) × (1 − V_j_)/V_j_); M_0_ = 4 × (F_300µs_ − F_0_)/(F_m_ − F_0_); and V_j_ = (F_2ms_ − F_0_)/(F_m_ − F_0_)

where M_0_ is the average value of the initial slope of the relative variable fluorescence of Chl *a*, which reflects the closing rate of the PSII reaction centers, and V_j_ is the relative level of fluorescence in phase J after 2 ms.

PAM fluorimetry (Junior-PAM, Walz, Germany) was used to evaluate the photosynthetic apparatus state. The values F_0_, F_v_, F_m_, F_m_′, and F′, as well as the PSII maximum (F_v_/F_m_) and effective Y(II) (F_m_′−F_t_)/F_m_′ photochemical quantum yields and non-photochemical quenching (NPQ) (F_m_/F_m_′−1), were determined. Here, F_m_ and F_m_′ are the maximum Chl fluorescence levels under dark- and light-adapted conditions, respectively. F_v_ is the photoinduced change in fluorescence, and F_t_ is the level of fluorescence before a saturation impulse is applied. F_0_ is the initial Chl fluorescence level. Actinic light was switched on for 10 min [I = 125 μmol (photons) m^−2^ s^−1^].

### 2.5. RNA Extraction and qRT-PCR

RNA isolation was performed according to the method of Kolosova et al. (2004) [25] with some modifications suggested by Pashkovskiy et al. (2019) [26]. The quantity and quality of the total RNA were determined using a NanoDrop 2000 spectrophotometer (Thermo Fisher Scientific, Waltham, MA, USA). cDNA synthesis was performed using the M-MLV Reverse Transcriptase Kit (Fermentas, Canada) and the oligo (dT) 21 primer for genes of nuclear coding and random 6 (Evrogen, Moscow, Russia) for chloroplast coding genes expression. The expression patterns of the genes were assessed using the CFX96 Touch™ Real-Time PCR Detection System (Bio-Rad, Hercules, CA, USA). The transcript levels were normalized to the expression of the *Actin 1* gene. The mRNA levels were expressed as a ratio of the corresponding values for the WFL plants. The relative gene-expression signal intensity at the WFL plants was considered to have a value of 1. Gene-specific primers (Table 1) for photosystem II protein D1 (*psbA*, ABO77179.1), cryptochrome 1 (*Cry1*, K7R334), cryptochrome 2 (*Cry2*, T2FFB6), phytochrome P (*phyP*, AIY54822.1), phytochrome N (*phyN*, AFV79519.1), phytochrome O (*phyO*, A7Y6Q6), phytochrome-interacting factor 3 (*PIF3*, D5ABG4), chalcone synthase (*CHS*, AF543757.1), stilbene synthase (*STS*, S50350.1), histidine-containing phosphotransfer 1 (*HPT1*, ALN42232.1), type-A response regulators (*RR-A*, FJ717710.1), and auxin-induced protein 1 (*Aux/IAA*, AY289600.1) were selected using nucleotide sequences from the National Center for Biotechnology Information (NCBI) database (Available online: http://www.ncbi.nlm.nih.gov (accessed on 15 May 2021)) and database www.uniprot.org (Available online: http://www.uniprot.org (accessed on 20 May 2021)) with Vector NTI Suite 9 software (Invitrogen, Carlsbad, CA, USA).

### 2.6. Histochemical Studies of the Hypocotyls

Anatomical studies were performed using light microscopy methods on live preparations of cross-sections of hypocotyls of *P. sylvestris* seedlings prepared immediately before the study. Sections 10–30 µm thick were prepared using an HM650 V vibrating blade microtome (Thermo Fisher Scientific, Waltham, MA, USA) through the central part of the organ. The obtained sections were stained with 3% phloroglucinol/HCl reagent (Sigma, P3502) for 2 min and then washed with phosphate buffered saline (70 mM, pH = 7.4) [27]. The sections were photographed under an Imager D1 light microscope (Carl Zeiss, Oberkochen, Germany) using a Levenhuk M800 PLUS digital photo attachment with a resolution of 8.0 MPI (Levenhuk, Tampa, FL, USA). The resulting images were processed with the public domain software ImageJ v.1.49 (NIH; http://rsb.info.nih.gov/ij). The diameter of the hypocotyl, the number of lignified xylem cells, the sectional area of the hypocotyl, the area of the xylem, and the average sectional area of one xylem cell were determined on the sections of the hypocotyls.

### 2.7. Statistics

The number of biological replicates for the determination of the fresh biomass of the roots and needles ranged from 27 to 39; 13 biological replicates were performed for the histochemical studies of the hypocotyls, and 6 biological replicates were used for photosynthetic and respiration rates as well as photochemical activity. Each plant sample fixed in liquid nitrogen was treated as a biological replicate; therefore, there were six biological replicates for the pigment contents and gene expression analyses.

The data were statistically analyzed using SigmaPlot 12.3 (Systat Software, San Jose, USA) with one-way analysis of variance (ANOVA) followed by Duncan’s method for normally distributed data (in the figures, significant differences are denoted by different capital letters). Kruskal–Wallis one-way ANOVA was performed on ranks followed by a Student-Newman–Keuls post hoc test or by a Dunn’s post hoc test for non-normally distributed data and data with unequal variance (in the figures, significant differences are denoted by different italic letters for the Student-Newman–Keuls post hoc test or by different boldface italic type for the Dunn’s post hoc test). Different letters were used to indicate significance at *p* < 0.05. The values presented in the tables and figures are the arithmetic means ± standard errors.

## 3. Results

### 3.1. Growth and Morphological Parameters

**Figure 2:**
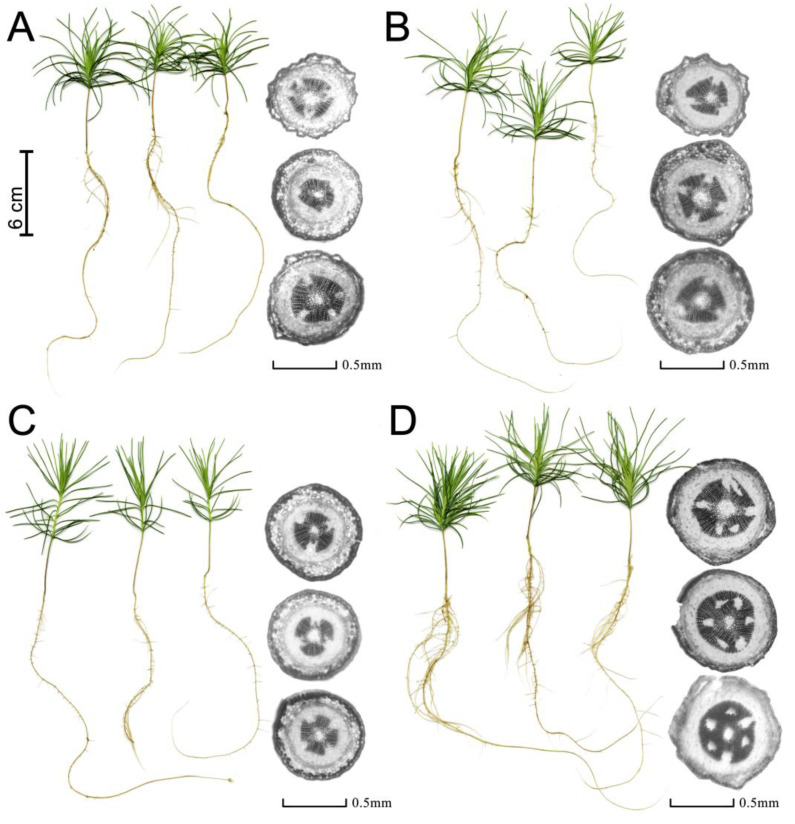
The morphological changes of *P. sylvestris* seedlings with dependence on light quality. WFL (**A**), BRL (**B**), BL (**C**), and RL (**D**).

Narrowband light caused marked changes in plant morphology and growth (Figure 2).

**Figure 3:**
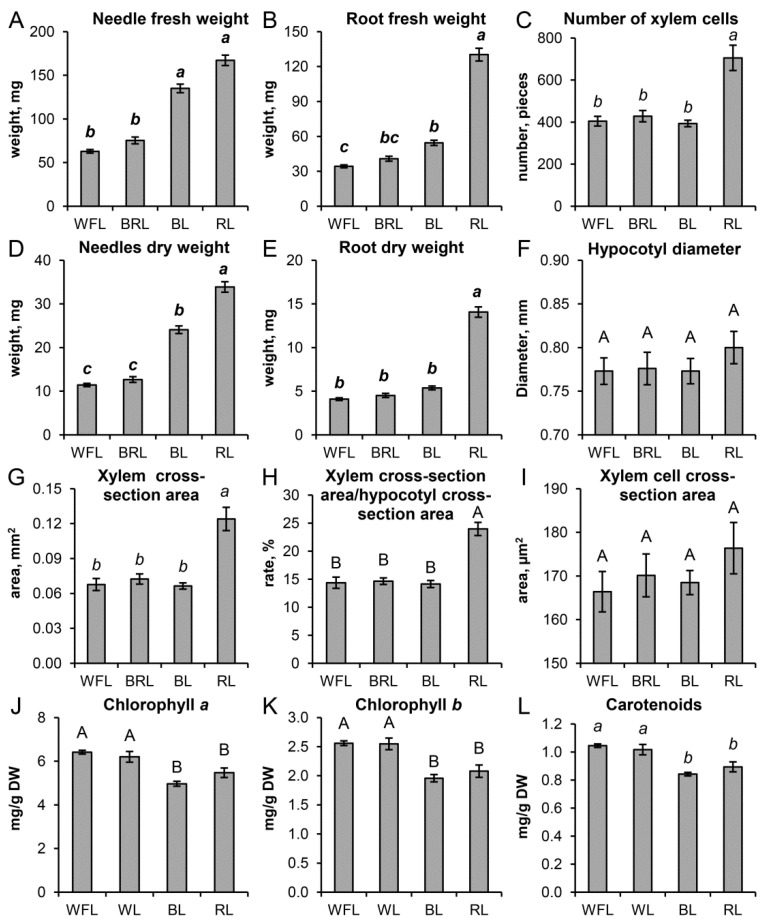
Effect of light quality on the needle (**A**) and root (**B**) fresh weight (mg); number of xylem cells (**C**) (pieces); needle (**D**) and root (**E**) dry weight (mg, DW); hypocotyl diameter (**F**) (mm); xylem cross-section area (**G**) (mm); xylem cross-section area and hypocotyls cross-section area ratio (**H**) (%); xylem cell cross-section area (**I**) (µm^2^); and content of Chl *a* (**J**) mg/g DW, Chl *b* (**K**) mg/g DW, and total carotenoids (**L**) mg/g DW in *P. sylvestris* seedlings. Values are the mean ± SE. Different capital letters denote statistically significant differences in the means at *p* < 0.05 (ANOVA followed by Duncan’s method). Different italic letters denote statistically significant differences in the means at *p* < 0.05 (Kruskal–Wallis ANOVA of the ranks followed by the Student-Newman-Keuls post hoc test). Different boldface italic letters denote statistically significant differences in the means at *p* < 0.05 (Kruskal–Wallis ANOVA of the ranks followed by the Dunn’s method).

Thus, RL increased the weight of the needles by 2.6 times, and BL increased the weight of the needles by 2.1 times relative to the WFL taken as a control (Figure 3A). BL caused hypocotyl elongation, and plants with RL formed short, larger plants. In addition, plant root weights in the RL variant were 3.8 times higher than the root weight in the control (WFL) and, on average, 2.7 times higher than in the other experimental variants (Figure 3B). Moreover, there was an increase in both the fresh and dry weight of the root in RL (Figure 3B,E) and dry weight of needles in BL and RL in relation to WFL control (Figure 3D). In general, RL led to a more intensive development of the root system of the seedlings and an increase in the number of lateral roots of the first and second orders (Figure 2). Under the influence of RL, in the hypocotyls of the seedlings, 1.7 times more xylem cells were formed compared to the other variants of the experiment (Figure 3C), in the absence of any differences in the total area of the hypocotyls (Figure 3F). It is important to note that the growth of xylem under RL was not accompanied by thickening of the hypocotyls (Figure 3F), but the area of the xylem increased relative to other tissues (Figure 3G,H), while the diameter of xylem cells did not differ significantly (Figure 3I).

### 3.2. Contents of Photosynthetic Pigments

When grown on a narrowband RL and BL, a reduced content of chlorophylls and carotenoids was observed (by an average of 10–20%), while the BRL and WFL variants were comparable (Figure 3J–L).

### 3.3. Fluorescence Parameters and CO_2_ Gas Exchange

The photosystem II (PSII) maximum quantum yield (F_v_/F_m_) was approximately 0.81 and did not depend on the light quality used in the experiments (Table 2).

The PSII effective quantum yield (Y(II)) was the lowest in BRL plants (0.44); at the same time, in BL and WFL plants, this indicator remained at a comparable level and amounted to approximately 0.51. The highest value of the Y(II) parameter was observed when growing seedlings under RL (0.57) (Table 2). The non-photochemical quenching (NPQ) parameter was the smallest in RL plants. When plants were grown on BL and WFL, NPQ was comparable and amounted to approximately 0.90; the highest NPQ value (1.67) was observed in the BRL plants (Table 2).

The intensity of CO_2_ gas exchange did not differ noticeably between the samples. The largest value of the parameter P_n_ (16.3 µmol CO_2_ m^−2^ s^−1^) was observed in the BRL variant, and the smallest value was 12.1 µmol CO_2_ m^−2^ s^−1^ in the RL variant, while the photosynthesis rate in the seedlings under the WFL and BL conditions was intermediate (Table 2).

The respiration rate (R) in the WFL and BRL variants was the highest (approximately 8.8 µmol CO_2_ m^−2^ s^−1^), while in BL and RL plants, this value was reduced to 5.1 and 2.1 µmol CO_2_ m^−2^ s^−1^, respectively. As a consequence, the calculated parameters of the R/P_n_ ratio and the P_n_–R carbon balance changed accordingly. The respiration/photosynthesis ratio was the highest in the WFL and BRL variants, lower in the BL variant, and the lowest in the RL plants (0.17). At the same time, the highest value of the carbon balance, assessed by the difference between the rates of photosynthesis and respiration, was observed in the RL variant (Table 2).

The PSII performance index (PI_ABS_) was on average 1.5 times higher in the RL variant than in the other variants. At the same time, all other options did not differ significantly among themselves (Table 2).

### 3.4. Gene Expression

**Figure 4:**
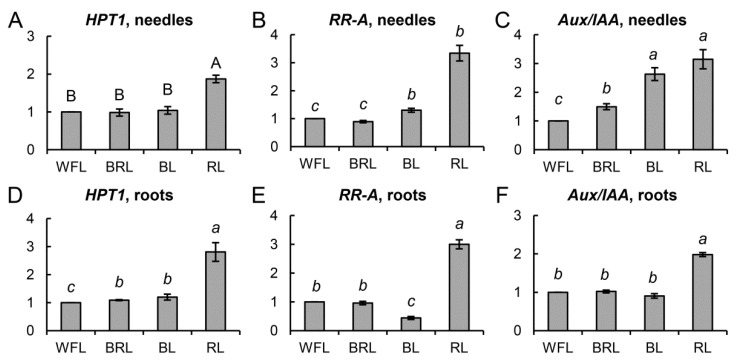
Effect of light quality on the transcript levels of different groups of genes in *P. sylvestris* seedlings. *HPT1, RR-A*, and *Aux/IAA* in needles (**A**–**C**) and roots (**D**–**F**). The mRNA levels of the genes were expressed as the BRL, BL, RL/WFL ratio (fold change BRL, BL, RL/WFL). Values are the mean ± SE. Different capital letters denote statistically significant differences in the means at *p* < 0.05 (ANOVA followed by Duncan’s method). Different italic letters denote statistically significant differences in the means at *p* < 0.05 (Kruskal–Wallis ANOVA of the ranks followed by the Student-Newman-Keuls post hoc test).

The transcript level of genes involved in hormonal signaling of cytokinins and auxins significantly changed in the variants of narrow-band light. Expression of the *HPT1* gene in the needles of RL plants was increased almost two times, and in the roots, it was 2.7 times higher than that in the WFL control (Figure 4A,D).

At the same time, the level of *RR-A* transcription in the RL variant was 3.3 and 3.0 times higher than that in WFL needles (Figure 4B) and roots (Figure 4E), respectively. In contrast, in the roots of the BL variant, *RR-A* expression was more than 2-fold lower than that in the WFL control (Figure 4E). In the needles of the seedlings, the expression of the *Aux/IAA* gene in the RL and BL plants was, on average, 2.8 times higher than that in the WFL control (Figure 4C). At the same time, in the roots, the level of *Aux/IAA* transcripts was higher only in the RL variant (Figure 4F).

**Figure 5:**
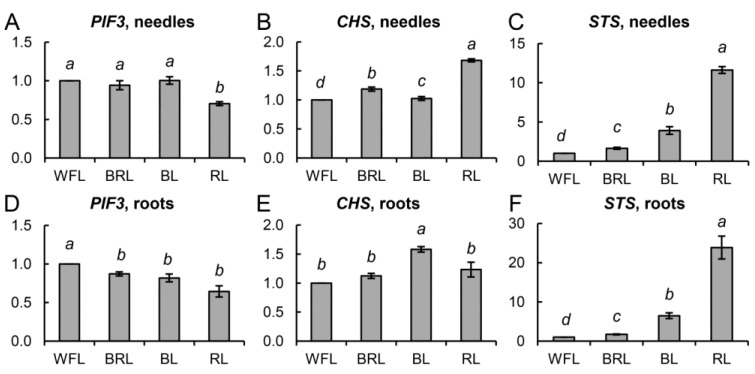
Effect of light quality on the transcript levels of different groups of genes in *P. sylvestris* seedlings. *PIF3, CHS*, and *STS* in needles(**A**–**C**), and roots (**D**–**E**). The mRNA levels of the genes were expressed as the BRL, BL, RL/WFL ratio (fold change BRL, BL, RL/WFL). Different italic letters denote statistically significant differences in the means at *p* < 0.05 (Kruskal–Wallis ANOVA of the ranks followed by the Student-Newman–Keuls post hoc test).

We also studied the transcription of genes responsible for light signaling and the synthesis of secondary metabolites, such as the transcription factors *PIF3*, *CHS*, and *STS*. The expression level of the *PIF3* gene in the RL variant compared to that of WFL was reduced in needles by 30% (Figure 5A), while that of the *CHS* gene, on the contrary, increased 1.5 times compared to those of BRL and WFL (Figure 5B).

An increase in the transcript level of the *STS* gene was also observed more than 11 times in needles (Figure 5C) and more than 23 times in roots (Figure 5F) in the RL variant, and approximately 4 times in needles and 6 times in roots in the BL variant (Figure 5C,F).

**Figure 6:**
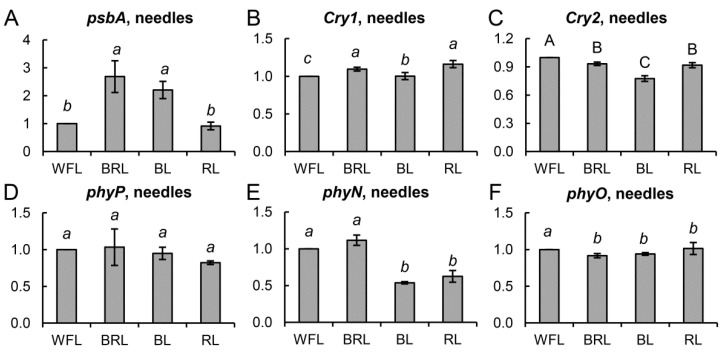
Effect of light quality on the transcript levels of different groups of genes in *P. sylvestris* needles. *psbA* (**A**), *Cry1* (**B**), and *Cry2* (**C**); and *phyP* (**D**), *phyN* (**E**), and *phyO* (**F**). The mRNA levels of the genes were expressed as the BRL, BL, RL/WFL ratio (fold change BRL, BL, RL/WFL). Values are the mean ± SE. Different capital letters denote statistically significant differences in the means at *p* < 0.05 (ANOVA followed by Duncan’s method). Different italic letters denote statistically significant differences in the means at *p* < 0.05 (Kruskal–Wallis ANOVA of the ranks followed by the Student-Newman–Keuls post hoc test).

Over the course of the experiment, the gene expression of the main proteins of photosystems I and II was studied. Among the large number of analyzed genes (*psbA,B,C,D,S; petA,C,D,E; psaA,B; flvA,B; Lhc1,2*), only the transcription levels of *psbA* encoding the PSII key protein D1 changed significantly and reliably. Thus, in variants BRL and BL, a twofold increase in the level of *psbA* transcripts was observed, while RL did not cause changes in the expression of this gene (Figure 6A).

In addition, the gene expression of apoproteins of the main blue and red light photoreceptors was studied (Figure 6B–D,F). Significant differences were observed in the *phyN* gene transcripts, the level of which was reduced by almost 2-fold in BL and RL relative to the WFL control (Figure 6E). Significant but negligible differences were observed in the transcript levels of *Cry1* and *Cry2* genes (Figure 6B,C).

## 4. Discussion

Seedlings of *P. sylvestris* are photophilous; however, in the first few seasons of their life, they can only be higher than the surrounding herbaceous plants for a short period of time, which causes a lack of light, primarily in the red and blue spectral ranges. It is known that RL, in contrast to BL, has a significant effect on the stem growth, stem diameter, and size and dry weight of *Picea abies* needles [20]. Thus, in contrast to growing under BL and WFL, RL increased the biomass and stem diameter of *Brassica oleracea* plants [28]. At the same time, photosynthesis in RL plants was noticeably higher than in other variants, which probably led to an increasing biomass [28]. In our work, RL influenced the morphology of *P. sylvestris* seedlings, which was manifested in an increase in the mass of the root system by more than 3.8 times, complications of root branching, and an increase in the mass of the needles by 2.6 times in comparison with WFL. At the same time, RL caused a slight decrease in CO_2_ gas exchange, as well as a significant (more than four times) decrease in respiration intensity relative to those of the WFL control (Table 2). This indicates a greater assimilation of carbon in RL plants. The slight difference in the pigment content is consistent with the fact that their photosynthetic apparatus is not dependent on light.

Regarding photochemical processes, the effective PSII quantum yield Y(II) was the highest in RL plants but the value of NPQ was small. We suppose that the increased Y(II) and complementary decreased NPQ are due to preferential excitation of photosystem I (PSI) by RL with a wavelength >685 nm. The contribution of the long-wave light is quite significant in red LEDs spectrum (Figure 1). Such light can lead to faster re-oxidization of the plastoquinone pool and reopening of PSII reaction centers [29]. As a result, reaction centers use absorbed light more efficiently.

Gymnosperms have the ability to synthesize chlorophyll in the dark due to the presence of the three genes of light-independent protochlorophyllide oxidoreductase L, N, and B (*ChlL*, *ChlN*, and *ChlB*) involved in the light-independent reduction of protochlorophyllide to chlorophyllide [30]. This also confirms the presence of special light regulation in conifers, which makes them a unique object of research.

The features of development and metabolism in plants induced by light of different spectral composition are primarily mediated by changes in the expression of light-dependent genes [20,30], including those encoding chloroplast proteins, photoreceptor apoproteins, transcription factors, and enzymes involved in the biosynthesis of secondary metabolites [31] and phytohormone signaling. In our work, we showed that several genes involved in the response to RL or BL are expressed in different ways. For example, the level of transcription of the *psbA* gene of the PSII main protein D1 increased in BL and BRL plants but decreased in RL plants (Figure 6A). This response to RL, as well as the accumulation of *psbA* transcripts under BL and RL conditions, is a typical response for most flowering plants [32]. Unfortunately, we did not observe noticeable changes in the expression of photoreceptor genes, with the exception of *phyN* (Figure 6B–F); on the other hand, under conditions of prolonged exposure to light of different spectra, it is impossible to exclude the presence of a sufficient number of active forms of photoreceptors, as well as the presence of regulation at the level of light signaling. In another work, it was shown that *phyN* is able to respond to different ratios of BL and RL in Scots pine seedlings of different growing regions [6].

The quality of light influences photomorphogenesis, photosynthesis, and plant growth through appropriate photoreceptors [33,34]. However, no significant difference in the expression level of photoreceptor genes was found between the light variants BL and RL, which is consistent with previous studies performed on *A. thaliana* seedlings [35]. The gene expression profiles of *A. thaliana* plants grown under BRL, RL, and BL were similar in all variants, and a significant proportion of differentially expressed genes under BL were also induced under RL. This indicates that the expression of light-regulated genes in *P. sylvestris* is not a unique response to BL or RL and that light of different spectral composition is able to regulate metabolic patterns in a similar way through the regulation of light signaling genes.

Transcription factors play an important role in the regulation of photosynthetic apparatus sensitivity to light of different spectral composition, since they, together with photoreceptors, are involved in the transduction of both light and hormonal signals. Phytochrome signaling transcription factors (PIFs) are important negative regulatory proteins that can alter the expression of a number of associated genes [6,36]. We observed a 30% decrease in the expression of the *PIF3* gene in RL plants, which may be associated with the activation of light signaling at the level of transcription of the corresponding genes (Figure 5A,D). These results demonstrate that the mechanisms by which light of different spectral composition controls the growth of *P. sylvestris* may involve angiosperm light signaling pathways. Transcription factors, together with photoreceptors, can influence the expression of hormonal signaling genes. Thus, a direct link between cytokinin signaling and light was found in a study demonstrating the key role of A-type RR regulators of cytokinin signaling [37,38,39]. RL induces *RR-A* expression in a PHYB-dependent manner. Thus, the overexpressing *RR-A* leads to hypersensitivity to RL [37]. Later research showed an important role for RR-A in photomorphogenesis [40,41]. RR-As are able to interact with PHYB via the cytokinin receptor (AHK). In support of this, we observed an increased expression of one of the main proteins of cytokinin signaling transduction (*HPT1*) by more than two times in roots and needles in the RL variant (Figure 4A,D). In addition, the PIFs are also involved in phytochrome-mediated regulation of auxin signaling under RL conditions, since these TFs are able to bind to the promoter regions of the *Aux/IAA* genes. PIFs modulate plant growth by directly controlling the expression of auxin signaling genes [11]. We assume that this, to a certain extent, explains the greater number of xylem cells in hypocotyls in the RL variant and, as a consequence, a greater accumulation of plant biomass (Figure 4C,F).

Along with the growth and development of plants, changes in the spectral composition of light also affect secondary metabolism. Chalcone synthase, the first enzyme in the biosynthesis of flavonoids, is expressed in needles of RL plants and in roots of BL plants (Figure 5B,E). In our study, RL stimulated gene families associated with the biosynthesis of the flavonoids (*CHS*, *STS*). Stilbenes are a family of polyphenolic secondary metabolites that act as phytoalexins [42,43]. Previously, it was shown that treatment with RL suspension culture of grape cells increased the biosynthesis of stilbenes [44,45]. In our study, we observed an increase in *STS* expression in roots and needles by more than 3.5 times in BL plants and more than 10 times in RL plants (Figure 5C,F). It can be assumed that, as in grape plants, stilbenes are involved in the photoadaptation of *P. sylvestris* plants to narrow-band RL and BL.

## 5. Conclusions

In this work, we tried to answer the question of what spectral range of light can be most favorable for the growth of *P. sylvestris* seedlings. It was shown that the RL spectral range is most favorable for growing *P. sylvestris* seedlings in a hydroculture, which manifested itself in both a greater mass of aboveground and underground organs and in an increase in the number of xylem cells. We found an increase in the level of transcripts of genes for auxin and cytokinin signaling (*HPT1, RR-A*, and *Aux/IAA*) and a decrease in the expression of TF *PIF3*, which in turn could activate the expression of a number of genes associated with the synthesis of secondary metabolites (*CHS, STS*). Based on the data obtained, we assumed that the large biomass of *P. sylvestris* plants under the RL might be due to a large accumulation of carbon in the needles, which corresponds to a better balance between photosynthesis and respiration, as well as to the increased activity of cytokinins and auxins in seedlings. Although *P. sylvestris* seedlings are able to grow in conditions of low RL content at the beginning of ontogenesis, better RL radiation can significantly improve their growth and development. The obtained results can serve as a basis for the development of a technology for the accelerated cultivation of planting material during reforestation when growing seedlings under artificial lighting, and they can also be used in biotechnology.

## Figures and Tables

**Table 1:** The primers for qRT-PCR analysis.

	Gene Bank ID	Gene Description	Plant	Gene	Primer 5′-3′	
					Forward	Reverse
1	ALN42232.1 (uniprot.org)	Histidine-containing phosphotransfer 1	*Pinus pinaster*	*HPT1*	GCTCAAGTATAGGAGCGCGG	CCAGCTTGTTTTTCACGAGGT
2	FJ717710.1 (ncbi.nlm.nih.gov)	Type-A Response Regulators	*Pinus pinea*	*RR-A*	CAGAAGGCGCTCAAGAGTTT	TTGTTGGTCCCTGGATCTTC
3	AY289600.1 (ncbi.nlm.nih.gov)	Auxin-induced protein 1 (IAA1)	*Pinus taeda*	*Aux/IAA*	GCCACCTGTCAAAGATTTCAG	TGAGGTCCACCTTTCTGAGA
4	ABO77179.1 (uniprot.org)	Photosystem II protein D1	*Pinus sylvestris*	*psbA*	TGAAGGTTACAGATTCGGTCA	TGAATATGCAACAGCAATCCA
5	K7R334 (uniprot.org)	Cryptochrome 1	*Pinus sylvestris*	*Cry1*	TATGGTGCACAGGGCAGATG	AAGCTGCAGAAGCTGTTCCT
6	T2FFB6 (uniprot.org)	Cryptochrome 2	*Picea abies*	*Cry2*	TTCCCTGGCTGCAACAGAAA	CCCAACATTGCTAGGCAGGA
7	AIY54822.1 (uniprot.org)	Phytochrome P	*Pinus sylvestris*	*phyP*	GGCATGTCCCTTGTTCAGGA	CTTCTGTGGGCCAAAGGTCT
8	AFV79519.1 (uniprot.org)	Phytochrome N	*Pinus sylvestris*	*phyN*	GGCTCAGAGGAGGACAAAGG	TTCTGCCCGGTCACATCTTG
9	A7Y6Q6 (uniprot.org)	Phytochrome O	*Pinus sylvestris*	*phyO*	AGATGTGACGTGGCAAAGGA	TGCGGGATTCCACTCAGAAC
10	D5ABG4 (uniprot.org)	Phytochrome-interacting factor 3	*Picea sitchensis*	*PIF3*	ATCAGCACTTCCTGGTTCCG	CAGGCTGAGTTGTTCCAGGT
11	AF543757.1 (ncbi.nlm.nih.gov)	Chalcone synthase	*Pinus uliginosa*	*CHS*	ATGGCTGCAGGAATGATGAAGG	AGTGCCAATAGCGAGGATG
12	S50350.1 (ncbi.nlm.nih.gov)	Stilbene synthase	*Pinus sylvestris*	*STS*	TCCGACTGGAACAAGTTGTTC	GCTTGGCCTCCACCCGATCAAG
13	CBB44933.1 (uniprot.org)	Actin 1	*Pinus sylvestris*	*Act1*	TTAGCAACTGGGATGACATGGA	CCTGAATGGCAACATACATAGCA

**Table 2:** Effect of light quality on the net photosynthetic and respiration rates, R/P_n_ ratio, (P_n_–R) difference, PSII maximum quantum yield (F_v_/F_m_), effective quantum yield Y(II), performance index PSII (PI_ABS_), and nonphotochemical fluorescence quenching (NPQ) in 6-week-old *P. sylvestris* seedlings. Values are the mean ± SE. Different normal-type letters denote statistically significant differences in the means at *p* < 0.05 (ANOVA followed by Duncan’s method).

Parameter	WFL	BRL	BL	RL
P_n_, µmol CO_2_ m^−2^ s^−1^	14.4 ± 2.1 ^ab^	16.3 ± 0.2 ^a^	13.7 ± 0.3 ^b^	12.1 ± 0.3 ^c^
R, µmol CO_2_ m^−2^ s^−1^	8.7 ± 0.8 ^a^	8.8 ± 0.1 ^a^	5.1 ± 0.2 ^b^	2.1 ± 0.1 ^c^
R/P_n_	0.62 ± 0.12 ^a^	0.54 ± 0.06 ^a^	0.37 ± 0.02 ^b^	0.17 ± 0.01^c^
(P_n_ – R), µmol CO_2_ m^−2^ s^−1^	5.7 ± 0.5 ^d^	7.5 ± 0.2 ^c^	8.6 ± 0.1^b^	10.0 ± 0.2 ^a^
F_v_/F_m_	0.814 ± 0.005 ^a^	0.803 ± 0.001 ^a^	0.815 ± 0.007 ^a^	0.812 ± 0.006 ^a^
PI_ABS_	7.73 ± 0.39 ^b^	7.85 ± 0.64 ^b^	6.45 ± 0.82 ^b^	10.21 ± 0.59 ^a^
Y_II_	0.52 ± 0.01 ^b^	0.44 ± 0.03 ^c^	0.50 ± 0.01 ^b^	0.57 ± 0.01 ^a^
NPQ	0.86 ± 0.10 ^b^	1.67 ± 0.21 ^a^	0.95 ± 0.12 ^ab^	0.66 ± 0.17 ^b^

## Data Availability

The data presented in this study are available on request from the corresponding authors. The data are not public.
